# Why Does the SARS-CoV-2 Delta VOC Spread So Rapidly? Universal Conditions for the Rapid Spread of Respiratory Viruses, Minimum Viral Loads for Viral Aerosol Generation, Effects of Vaccination on Viral Aerosol Generation, and Viral Aerosol Clouds

**DOI:** 10.3390/ijerph18189804

**Published:** 2021-09-17

**Authors:** Byung Uk Lee

**Affiliations:** Aerosol and Bioengineering Laboratory, Konkuk University, 120 Neungdong-ro, Gwangjin-gu, Seoul 05029, Korea; leebu@konkuk.ac.kr

**Keywords:** COVID-19, bioaerosol, aerosol transmission, severe acute respiratory syndrome coronavirus 2, virus transmission, airborne transmission, SARS-CoV-2 bioaerosol, air infection, viral infection, nosocomial infection, respiratory particle, contagious disease, Alpha variant, Delta variant, B.1.1.7, B.1.617.2, SARS-CoV-2 variant of concern, viral cloud, viral aerosol cloud

## Abstract

This study analyzes the reasons the severe acute respiratory syndrome coronavirus 2 (SARS-CoV-2) Delta variant of concern (VOC) spreads so rapidly. Novel topics such as universal conditions for the rapid spread of respiratory viruses, minimum viral loads for viral aerosol generation, effects of vaccination on viral aerosol generation, and viral aerosol clouds were studied. The analyses were based on experimental results and analytic model studies. Four universal conditions, namely asymptomatic host, high viral load, stability of viruses in air, and binding affinity of viruses to human cells, need to be satisfied for the rapid spread of respiratory viruses. SARS-CoV-2 and its variants such as the Alpha VOC and Delta VOC satisfy the four fundamental conditions. In addition, there is an original principle of aerosol generation of respiratory viruses. Assuming that the aerosol–droplet cutoff particle diameter for distinguishing potential aerosols from earthbound respiratory particles is 100 μm, the minimum viral load required in respiratory fluids to generate viral aerosols is ~10^6^ copies mL^−1^, which is within the range of the reported viral loads in the Alpha VOC cases and the Delta VOC cases. The daily average viral loads of the Delta VOC in hosts have been reported to be between ~10^9^ copies mL^−1^ and ~10^10^ copies mL^−1^ during the four days after symptom onset in 1848 cases of the Delta VOC infection. Owing to the high viral load, the SARS-CoV-2 Delta VOC has the potential to effectively spread through aerosols. COVID-19 vaccination can decrease aerosol transmission of the SARS-CoV-2 Alpha VOC by reducing the viral load. The viral load can explain the conundrum of viral aerosol spreading. The SARS-CoV-2 Delta VOC aerosol clouds have been assumed to be formed in restricted environments, resulting in a massive numbers of infected people in a very short period with a high spreading speed. Strong control methods against bioaerosols should be considered in this SARS-CoV-2 Delta VOC pandemic. Large-scale environmental monitoring campaigns of SARS-CoV-2 Delta VOC aerosols in public places in many countries are necessary, and these activities could contribute to controlling the coronavirus disease pandemic.

## 1. Viral Load Analysis for Aerosol Generation of Respiratory Viruses and the Original Principle for Viral Aerosol Generation

There is an original principle for aerosol generation of respiratory viruses. Smaller respiratory particles among various sized respiratory particles can be airborne for longer periods and water on their surfaces has a shorter evaporation time [1,2].Therefore, if sufficiently small virus-carrying respiratory particles are released from hosts into ambient air, viruses inside the particles transform into aerosol nuclei (viral aerosols) after water evaporation. The aforementioned original principle is the basis for the minimum viral loads required to generate sufficiently small virus-carrying respiratory particles (for viral aerosols). If the aerosol–droplet cutoff particle diameter distinguishing potential aerosols from earthbound respiratory particles (droplets = earthbound respiratory particles) is 100 μm [3], the minimum viral load required in respiratory fluids for generating the sufficiently small virus-carrying respiratory particles would be ~10^6^ copies mL^−1^ (virus size, 0.09 μm). This theoretical analysis on minimum viral loads for viral aerosol generation is based on the assumptions of a homogeneous distribution of viruses in respiratory fluids, considering one gene copy as a single virion, and a spherical volume ratio model for both respiratory particles and viruses (Supplementary Material Section) [4]. These assumptions can be considered as a limitation of this theoretical analysis. If the aerosol–droplet cutoff values distinguishing potential aerosols from earthbound particles are 50 μm, 10 μm, and 5 μm, the minimum viral loads required for generating viral aerosols are ~10^7^ copies mL^−1^, ~10^9^ copies mL^−1^, and ~10^10^ copies mL^−1^, respectively (Table 1).

**Figure 1:**
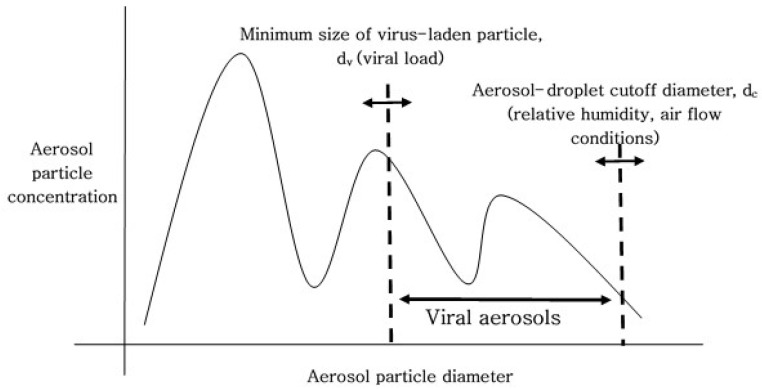
Size distribution of emitted respiratory particles. Generation of viral aerosols is determined by the minimum size of respiratory particles carrying viruses (d_v_, decided by a viral load) and the aerosol–droplet cutoff particle diameter (d_c_, which depends on the relative humidity and air flow conditions).

The aerosol–droplet cutoff particle diameter for respiratory particles depends on the relative humidity and air flow conditions. This analytic study demonstrates that higher viral loads in respiratory fluids decrease the minimum size of virus-laden respiratory particles, therefore generation of viral aerosols is induced by the higher viral loads [4]. As shown in Figure 1, generation of viral aerosols is determined by the minimum size of respiratory particles carrying viruses (d_v_, which is determined by the viral load) and the aerosol–droplet cutoff particle diameter (d_c_, which depends on the relative humidity and air flow conditions) (Figure 1) [4]. The minimum size of respiratory particles carrying viruses (d_v_) decreases (d_v_ moves to the left, in Figure 1) under high viral load conditions [4]; consequently, more viral aerosols can be generated under high viral load conditions.

## 2. Four Universal Conditions Required for the Rapid Spread of Respiratory Viruses, including SARS-CoV-2 and Its Variants (the Alpha VOC and the Delta VOC), and the Effect of Vaccination on Viral Aerosol Generation

In the coronavirus disease (COVID-19) pandemic, questions are arising regarding the underlying mechanisms responsible for the rapid spread of severe acute respiratory syndrome coronavirus 2 (SARS-CoV-2) and its variants such as the Alpha variant of concern (VOC) (formerly known as B.1.1.7) and the Delta VOC (formerly known as B.1.617.2). This study introduces the universal principles that explain the rapid spread of respiratory viruses. These principles apply to all contagious diseases caused by respiratory viruses.

There are four fundamental conditions that need to be satisfied for the rapid spread of respiratory viruses. The first is asymptomatic hosts with viral shedding that peaks during the early stage of infection. Symptomatic hosts (with fever, fatigue, and cough) have limited contact with other people because of self-recognition of infection. However, asymptomatic hosts interact with other people freely, and viruses can easily move from asymptomatic hosts to non-infected individuals. The second is massive shedding of the virus from the infected cells of hosts in their respiratory fluids, which is a high viral load condition. Small respiratory particles have a greater chance of carrying viruses (small virus-laden particles) under high viral load conditions than under low viral load conditions. Thus, viral aerosols can be generated from small virus-laden respiratory particles under high viral load conditions and spread in the nearby environment. The third is the stability of respiratory viruses in the outside air. For long distance transmission over a long period, the stability of airborne viruses is crucial. The fourth is a strong binding affinity of viruses to human cells at the receptors of entry points such as the nose, mouth, and eyes. The fundamental conditions can even facilitate each other, for example, if a virus has an extremely strong binding affinity to human cells, it can compensate for lower concentrations of the virus in the air under low viral load conditions. These four fundamental conditions are universal requirements for the rapid spread of respiratory viruses.

Concerning SARS-CoV-2, viral shedding by asymptomatic hosts has been reported to peak during the early stage of infection [5]. The viral load of SARS-CoV-2 in hosts reaches ~10^10^ copies mL^−1^ [6], which is significantly over the minimum viral load (~10^6^ copies mL^−1^) required for aerosol generation of respiratory viruses [4]. A study that investigated 282 clusters of infections found that a higher risk of transmission of SARS-CoV-2 was associated with high viral loads [6], and the high viral loads induced generation of viral aerosols [4]. Therefore, viral aerosols generated under high viral load conditions could contribute to the higher risk of transmission of the virus and generation of SARS-CoV-2 aerosols is natural because of the high viral loads. During the airborne stage, the stability of SARS-CoV-2 was found to be maintained [7,8]. SARS-CoV-2 aerosols, which were generated artificially inside a rotating drum, maintained their viability at 23 ± 2 °C and 53 ± 11% relative humidity conditions for 16 h [7]. SARS-CoV-2 has a strong affinity for human cells via angiotensin-converting enzyme 2 receptors [9].

Concerning the SARS-CoV-2 Alpha VOC (formerly known as B.1.1.7), viral concentrations in respiratory samples of hosts have been reported to range from ~10^7^ copies mL^−1^ to ~10^9^ copies mL^−1^ [10,11], which indicates that the viral variant has the potential to effectively spread through aerosols. In addition, viral loads of the Alpha VOC have been observed to be higher than those of the Wild-type (non-Alpha VOC) [12]. The SARS-CoV-2 Alpha VOC has been found to have ~two times greater affinity to human cells via angiotensin-converting enzyme 2 receptors than the Wild-type [13]. These can be persuasive explanations for the rapid spread of the SARS-CoV-2 Alpha VOC [14]. The above elucidation for the Alpha VOC and the spreading speed of the new Delta VOC (formerly known as B.1.617.2) in India and the United Kingdom [15] support that viral load of the Delta VOC in respiratory samples can be significantly high.

The SARS-CoV-2 Delta VOC has shown very high transmissibility [15], with high viral loads [16,17]. The SARS-CoV-2 Delta VOC has been shown to be less sensitive to neutralizing antibodies [18], resulting in a higher viral load in the host. The viral loads in individuals infected with the Delta VOC have been reported to be ~1260 times higher than those in individuals infected with the non-Delta variants in China [16]. The viral loads in individuals infected with the Delta VOC (on the first day after symptom onset) have been reported to be ~300 times higher than those in individuals infected with the Wild type cases in Korea [17]. The viral load of the Delta VOC in oropharyngeal swabs of hosts reached ~10^9^ copies mL^−1^ in China [16]. The daily average viral loads of the Delta VOC in hosts were reported to be between ~10^9^ copies mL^−1^ and ~10^10^ copies mL^−1^ during the four days after symptom onset (based on an investigation of 1848 cases of infection with the Delta VOC) in Korea [17]. These findings indicate that the Delta VOC has the great potential to effectively spread through aerosols.

Therefore, SARS-CoV-2 and its variants such as the Alpha VOC and the Delta VOC satisfy the four fundamental conditions for universal principles of rapidly spreading respiratory viruses.

It has been reported that vaccination reduced the viral loads in individuals infected with the SARS-CoV-2 Alpha VOC in the United Kingdom [12]. Therefore, vaccines have the ability to decrease the aerosol transmission possibility of the SARS-CoV-2 Alpha VOC. In addition, it has been reported that vaccination reduced the viral loads in individuals infected with SARS-CoV-2 in Israel [19,20].

The viral load principle for viral aerosol transmission is an important key in supporting that viral aerosols in the environment can be dominant factors in the COVID-19 issues. The viral loads and viral aerosols can explain the conundrum of viral spreading, including super-spreading events, during the pandemic [21,22,23,24,25].

## 3. Conclusions and Viral Aerosol Clouds

In Tehran in Iran, SARS-CoV-2 ribonucleic acid (RNA) has been detected in the air in public environments such as airports, subway stations, subway trains, and shopping centers [26]. It has been hypothesized that if viral loads are extremely high owing to inefficiency of immune systems against viral infections [16,17,18], this can enable the formation of viral aerosol clouds. Many viral aerosols emitted from a large number of infected individuals can form clouds of viral aerosols over the hosts, and these viral clouds can move around among people and spread the virus. Viral aerosol clouds are assumed to grow in size until all the surrounding people are infected, because viral aerosols from newly infected persons (hosts with viral shedding that peaks during the early stage of infection) contribute to viral clouds. In enclosed spaces, if several viral clouds merge, a large viral cloud can be generated. Large viral clouds can spread a large number of viruses to people in a short period of time, causing large-scale epidemics.

SARS-CoV-2 Delta VOC aerosol clouds are assumed to have been formed in India and the United Kingdom in 2021, based on the rapid spread and the large number of people infected within a short period [15]. Transmission via fomites and viral droplets cannot explain the current rapid spread of the Delta VOC. Mathematical models such as the well-known SIR model need to be revised to include the viral cloud hypothesis for the SARS-CoV-2 Delta VOC [27]. In outdoor environments, formation of viral aerosol clouds is expected to be less likely than that in indoor enclosed environments owing to dilution by ambient air. However, if many infected individuals are present in close proximity in outdoor environments, viral aerosol clouds can be assumed to be formed. It was reported that genetic materials of SARS-CoV-2 were detected in outdoor air aerosol samples (<0.8 copies m^−3^) in Italy [28].

Large-scale environmental monitoring of SARS-CoV-2 Delta VOC aerosols at many locations over a long period is necessary [24,26]. These measures could be useful for preventing new pandemics. In several hospitals, SARS-CoV-2 RNA has been detected in the air [29,30,31,32,33].

The universal principles of rapidly spreading respiratory viruses, which have been studied in this analysis, can be applied to all contagious diseases caused by respiratory viruses. The overwhelming fulfilment of the four fundamental conditions can lead to generation of viral clouds, which can result in rapid large-scale infections. Control methods against bioaerosols should be considered in such situations [34,35]. Face masks for reducing generation of viral aerosols and face-shields for eye-protection against viral aerosols should be considered as personal protective methods [25,35]. High efficiency filtering masks can be considered to protect non-infected individuals against viral aerosols in contaminated air environments [34,36]. Air filtration and ultraviolet irradiation in public facilities should also be considered in outbreak situations [34]. Viral aerosol clouds, minimum viral loads for the generation of viral aerosols, and strong control methods against bioaerosols should all be considered in the pandemic of respiratory viruses.

## Figures and Tables

**Table 1:** Minimum viral loads required for aerosol generation (the aerosol–droplet cutoff particle diameters to distinguish aerosols from droplets vary with the relative humidity and airflow conditions).

	Cutoff Particle Diameter to Distinguish Potential Aerosols from Earthbound Respiratory Particles	Minimum Viral Loads Required for Aerosol Generation
Minimum viral loads for aerosol generation (assumptions: homogeneous distribution; virus size 0.09 μm [sphere, d_virus_]; one gene copy = a single virion)	100 μm	~1.9 × 10^6^ copies mL^−1^
	50 μm	~1.5 × 10^7^ copies mL^−1^
	10 μm	~1.9 × 10^9^ copies mL^−1^
	5 μm	~1.5 × 10^10^ copies mL^−1^

## Data Availability

Not applicable.

## Supplementary Materials

The following are available online at https://www.mdpi.com/article/10.3390/ijerph18189804/s1.
