# Supplementary material for: Why Does the SARS-CoV-2 Delta VOC Spread So Rapidly? Universal Conditions for the Rapid Spread of Respiratory Viruses, Minimum Viral Loads for Viral Aerosol Generation, Effects of Vaccination on Viral Aerosol Generation, and Viral Aerosol Clouds
Source: Int J Environ Res Public Health. 2021 Sep 17;18(18):9804. doi: 10.3390/ijerph18189804 (PMC8470664; doi:10.3390/ijerph18189804)
Supplement: Supplementary file 1 [file ijerph-18-09804-s001.zip › ijerph-1366069-supplementary.pdf]

Supplementary material

(Lee, B.U. (2021). Why Does the SARS-CoV-2 Delta VOC Spread So Rapidly? - Universal Conditions for the Rapid Spread of Respiratory Viruses, Minimum Viral Loads for Viral Aerosol Generation, Effects of Vaccination on Viral Aerosol Generation, and Viral Aerosol Clouds. *Int. J. Environ. Res. Public Health*)

1. Analysis details

This theoretical analysis of the minimum viral load for viral aerosols is based on the assumptions of a homogeneous distribution of viruses in respiratory fluids, considering one gene copy as a single virion, and a spherical volume ratio model for both respiratory particles and viruses [1].

Assumption: (Virus size: 0.09 micrometer)

Analysis 1

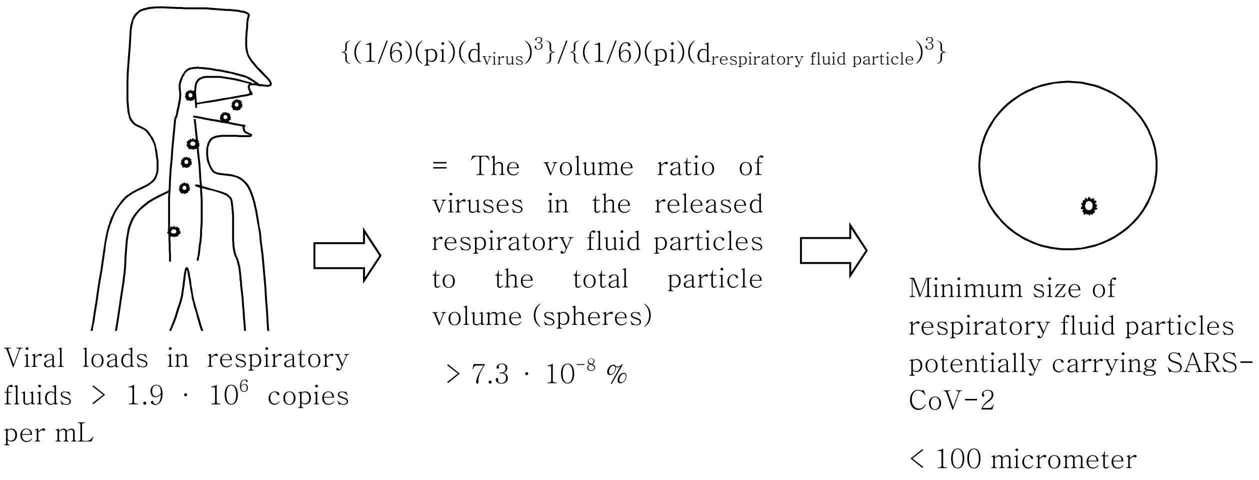

Supplementary-Figure 1. Minimum viral load required for aerosol transmission (assumptions: the aerosol-droplet cutoff particle diameter to distinguish aerosols from droplets for respiratory particles is assumed to be 100 micrometer; homogenous distribution; virus size = 0.09 micrometer [sphere, d_{virus}] assumption; one gene copy=a single virion)

$(1.9 \cdot 10^{6}$ copies$) / (1$ mL$)$

$= [1.9 \cdot 10^{6} \cdot \{(1/6)(pi)(0.09 \cdot 10^{-6}m)^3\}] / (10^{-6}m^3)$

$= \{(1/6)(pi)(0.09 \cdot 10^{-6}m)^3\} / \{(1/6)(pi)(d_{respiratory\ fluid\ particle})^3\}$

$d_{respiratory\ fluid\ particle}$=100 micrometer

References

1. Lee, B.U. Minimum sizes of respiratory particles carrying SARS-CoV-2 and the possibility of aerosol generation. *Int. J. Environ. Res. Public Health* 2020, 17, 6960, https://doi.org/10.3390/ijerph17196960.
